# Biological markers of oxidative stress: Applications to cardiovascular research and practice^☆^

**DOI:** 10.1016/j.redox.2013.07.006

**Published:** 2013-10-08

**Authors:** Edwin Ho, Keyvan Karimi Galougahi, Chia-Chi Liu, Ravi Bhindi, Gemma A. Figtree

**Affiliations:** a North Shore Heart Research Group, Kolling Institute of Medical Research, University of Sydney, Sydney, Australia; b Department of Cardiology, Royal North Shore Hospital, Sydney, Australia

**Keywords:** CVD, cardiovascular disease, ROS, reactive oxygen species, IsoP, isoprostane, MDA, malondialdehyde, TBARS, thiobarbituric acid reacting substance, O_2_^•−^, superoxide, ^•^OH, hydroxyl radical, HO_2_^•^, hydroperoxyl radical, ONOO^−^, peroxynitrite, HOCl, hypochlorous acid, H_2_O_2_, hydrogen peroxide, NO_2_, nitrogen dioxide, MPO, myeloperoxidase, OxLDL, Oxidized low-density lipoprotein, GSH, glutathione (reduced), Biomarker, Cardiovascular disease, Glutathionylation, Oxidative stress, Prognosis

## Abstract

Oxidative stress is a common mediator in pathogenicity of established cardiovascular risk factors. Furthermore, it likely mediates effects of emerging, less well-defined variables that contribute to residual risk not explained by traditional factors. Functional oxidative modifications of cellular proteins, both reversible and irreversible, are a causal step in cellular dysfunction. Identifying markers of oxidative stress has been the focus of many researchers as they have the potential to act as an “integrator” of a multitude of processes that drive cardiovascular pathobiology. One of the major challenges is the accurate quantification of reactive oxygen species with very short half-life. Redox-sensitive proteins with important cellular functions are confined to signalling microdomains in cardiovascular cells and are not readily available for quantification. A popular approach is the measurement of stable by-products modified under conditions of oxidative stress that have entered the circulation. However, these may not accurately reflect redox stress at the cell/tissue level. Many of these modifications are “functionally silent”. Functional significance of the oxidative modifications enhances their validity as a proposed biological marker of cardiovascular disease, and is the strength of the redox cysteine modifications such as glutathionylation. We review selected biomarkers of oxidative stress that show promise in cardiovascular medicine, as well as new methodologies for high-throughput measurement in research and clinical settings. Although associated with disease severity, further studies are required to examine the utility of the most promising oxidative biomarkers to predict prognosis or response to treatment.

## Highlights

• Oxidative stress is a common mediator in pathobiology of risk factors for CVD.
• Oxidative modifications of proteins and lipids alter cellular function.
• Some oxidative biomarkers have been associated with severity of CVD.
• Pathophysiologically relevant biomarkers may integrate the effect of risk factors.
• Utility of oxidative biomarkers to guide prognosis/treatment merits further work.

## Graphical abstract

**Figure:**
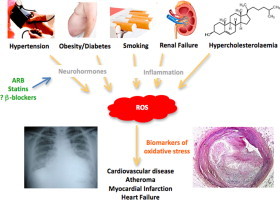

## Introduction

The term biomarker has been defined by The National Institutes of Health as “a characteristic that is objectively measured and evaluated as an indicator of normal biological processes, pathogenic processes, or pharmacological responses to a therapeutic intervention” [1]. Applications include diagnosis, prognosis and individualization of therapy in cardiovascular disease (CVD). Examples of circulating biomarkers that have been incorporated into clinical practice, and shown to have value in addition to traditional cardiovascular risk factor analysis, include N-terminal pro-B-type natriuretic peptide (NT-proBNP) for heart failure [2], glycated haemoglobin (HbA1c) for glycaemic control in diabetes [3], high-sensitivity troponin I [4] and high-sensitivity C-reactive protein (hs-CRP) for cardiovascular risk prediction [5]. Factors that determine the clinical utility of a biomarker include the ease and cost of measurement, its performance characteristics (e.g. sensitivity, specificity, etc.) and evidence for guiding management and improving patient outcome [6].

The most promising biomarkers are the ones that closely correlate with the pathophysiological process of the disease. The role of oxidative stress in the pathophysiology of CVD is well established [7,8]. Reactive oxygen species (ROS) are derived from many sources including mitochondria, xanthine oxidase, uncoupled nitric oxide synthases and NADPH oxidase [9]. In addition to generalized oxidation resulting in cell dysfunction, necrosis or apoptosis, ROS also induce specific post-translational modifications that alter the function of important cellular proteins and signalling pathways in the heart [10–13]. The important role of oxidative stress in cardiovascular pathophysiology has encouraged quantification of ROS as a promising biomarker reflecting the disease process. However, this has proven to be a complex challenge given the evanescent nature of ROS. The short half-life of these species makes them excellent signalling molecules but confounds their measurement in the circulation of complex biological systems by standard approaches such as spin-trapping [7]. Instead the focus has been on measuring stable markers in the circulation that may reflect systemic oxidative stress. This review will discuss current biomarkers of oxidative stress focusing on their advantages and disadvantages in research and clinical setting and future directions in this field.

## Biomarkers of oxidative stress

**Fig. 1:**
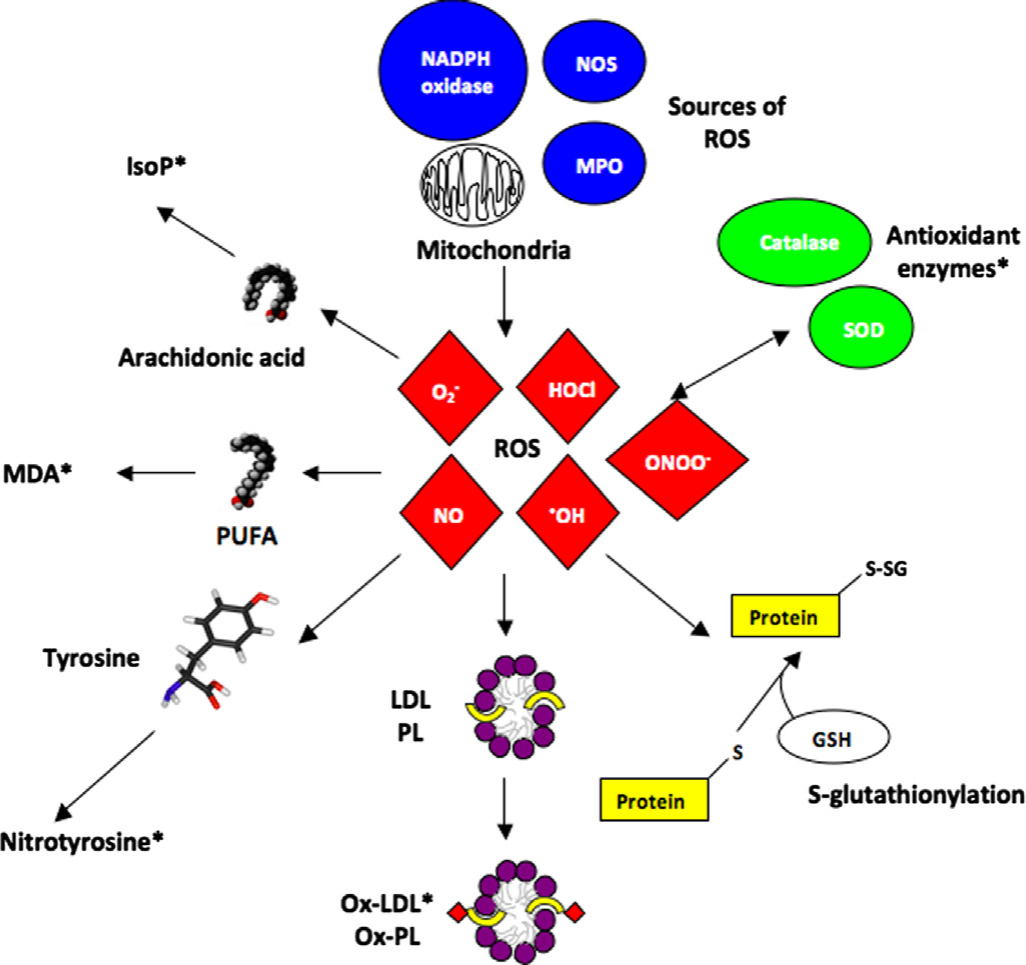
Formation pathways of selected biomarkers of oxidative stress. Biomarkers that have been shown to have prognostic significance in cardiovascular disease are marked with ⁎. GSH=glutathione (reduced), PUFA=polyunsaturated fatty acids, see text for other abbreviations.

**Fig. 2:**
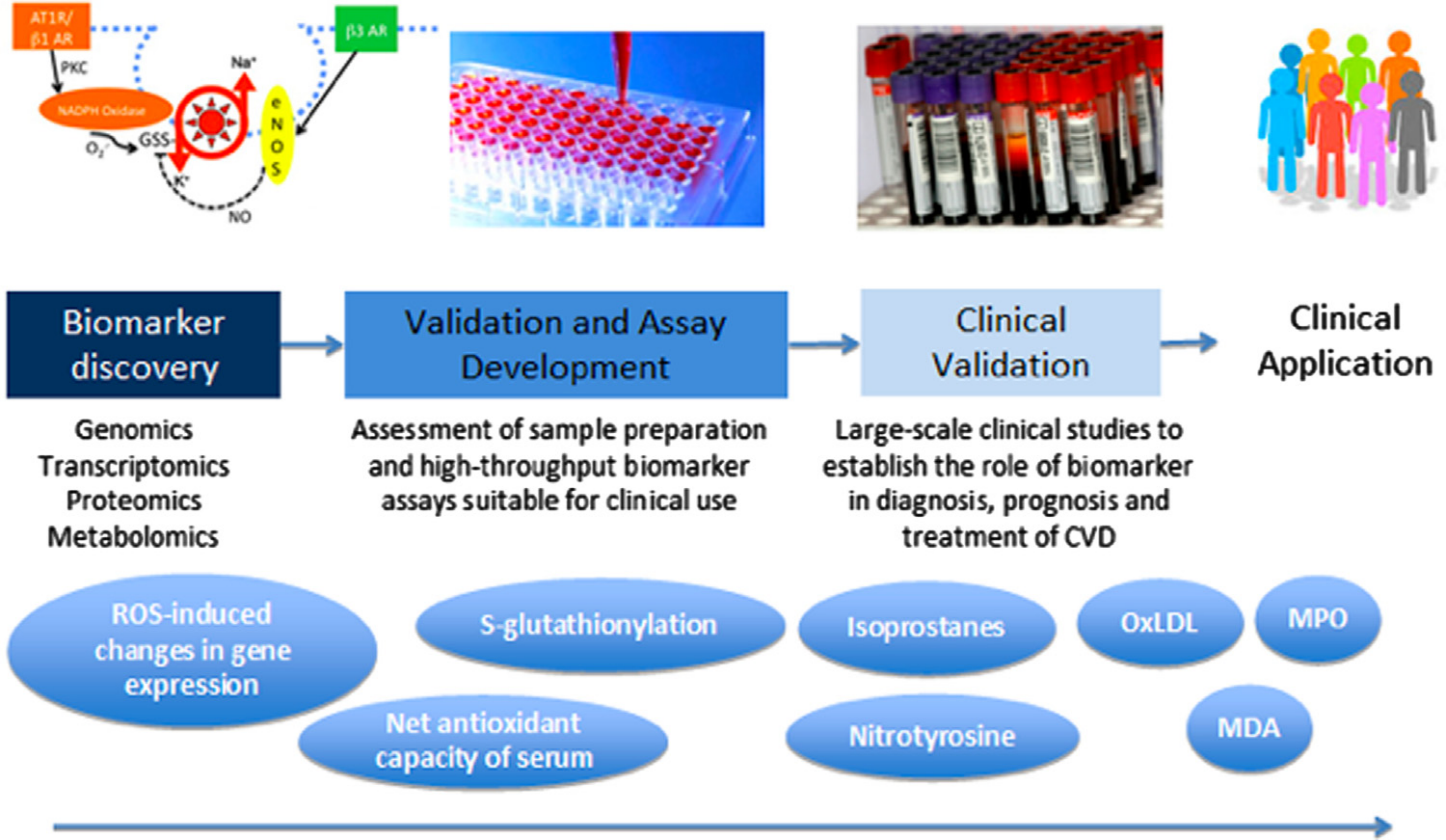
Schematic timeline of required steps in biomarker development, from discovery in the Laboratory to clinical application after validation in large scale clinical trials. Although many ROS biomarkers have reached clinical trials level, only few are regularly applied to patients in clinical practice.

Biomarkers of oxidative stress can be classified as molecules that are modified by interactions with ROS in the microenvironment; and molecules of the antioxidant system that change in response to increased redox stress. DNA, lipids (including phospholipids), proteins and carbohydrates are examples of molecules that can be modified by excessive ROS *in vivo*. This is shown schematically in Fig. 1. Of these modifications, some are known to have direct effects on function of the molecule (e.g. inhibit enzyme function), but others merely reflect the degree of oxidative stress in the local environment. The functional significance or causal role of the oxidative modification on cell, organ and system function is recognized as a key determinant of the validity of the marker. Other factors influencing the clinical applicability of a ROS biomarker include the ease of obtaining an appropriate biological specimen; the stability of the biomarker throughout various storage conditions and specimen preparation steps; and the specificity, sensitivity and reproducibility of the assay used to measure the modification [14]. Table 1 summarizes the advantages and disadvantages of the selected oxidative stress biomarkers discussed below. Fig. 2 demonstrates the timeline and required steps for biomarker development for clinical application.

## Lipid peroxidation

The important role of free radical oxidation of cellular components in CVD has been recognized since the proposal of the oxidative theory of atherogenesis [15,16]. Lipids are susceptible targets of oxidation because of their molecular structure abundant with reactive double bonds [17]. Two of the most well studied markers of lipid peroxidation are isoprostanes (IsoPs) and malondialdehyde (MDA). Other lipid oxidation products that have been explored as biomarkers include lipid hydroperoxides, fluorescent products of lipid peroxidation, oxidation resistance assays and oxysterols.

### Isoprostanes

IsoPs are a family of stable, prostaglandin-like compounds generated from the peroxidation of arachidonic acid, a polyunsaturated fatty acid present in phospholipids of cell membranes [18]. The generation of IsoPs from arachidonic acid is independent of the cyclooxygenase enzyme that catalyzes the formation of prostaglandins from arachidonic acid [19]. Sources of free radicals for IsoPs formation include: (1) mitochondrial electron transport chain (superoxide (O_2_^•−^) and hydroxyl radical (^•^OH)), (2) P450 enzymes (O_2_^•−^ and ^•^OH), (3) lipoxygenase (hydroperoxyl radical (HO_2_^•^)) and (4) transition-metal catalyzed formation of free radicals [20]. IsoPs are subsequently released from the cell membrane into circulation by phospholipases [21], and can then be quantified in tissues, blood and urine. F_2_-IsoPs, so called because they contain F-type prostane rings, are the most stable of the IsoPs family and show the most potential as a biomarker. The independence of circulating IsoPs levels of renal or hepatic function allows them to more directly reflect IsoPs production and oxidative stress [22].

IsoPs can be measured using gas chromatography–mass spectrometry (GC/MS), liquid chromatography–mass spectrometry (LC/MS), enzyme-linked immunosorbance assays (ELISA) and radioimmunoassay in plasma and urine samples [23]. Commercial immunoassay kits for IsoPs have been developed that are cheap and easy to use but have variable performance and results correlate poorly with mass spectrometric techniques [24], which are is still regarded as the gold standard for IsoP quantification [22]. *Ex vivo* stability is an important consideration when applying a biomarker to epidemiological studies. A small study revealed that the concentrations of F_2_-IsoPs in human plasma measured by GC/MS at 0 and 24 h *ex vivo* were similar, but significant *ex vivo* artefactual generation of F_2_-IsoPs occurred in plasma stored on ice for 36 h [25]. This is presumably secondary to on-going oxidation of lipids in plasma sample during prolonged storage and suggests that the utility of plasma F_2_-IsoPs is limited by time to analysis. No study of stability of urinary F_2_-IsoPs has been published to our knowledge.

Levels of IsoPs in plasma and urine samples have been shown to correlate with *in vivo* oxidative stress in a number of animal and human studies [20,26]. IsoPs are elevated in association with risk factors such as cigarette smoking, hypercholesterolaemia, diabetes mellitus, obesity, and hyperhomocysteinemia [22], as well as myocardial ischaemia/reperfusion [27]. Elevated F_2_-IsoPs occurring in rhabdomyolysis as a result of redox cycling between ferric and ferryl forms of myoglobin, plays a causal role in renal vasoconstriction and associated renal failure [28], F_2_-IsoP levels are increased in human atherosclerotic lesions compared with normal vascular tissue [29], and may participate in the actual pathogenesis of atherosclerosis through effects on vasoconstriction, platelet aggregation, and proliferation of vascular smooth muscle cells (VSMCs) [19,26]. Clinical trials attempting to decrease F_2_-IsoP production in humans with antioxidant therapy, such as vitamin C and/or E supplementation, have generated mixed results [30]. It is also not known if reduction of IsoP levels correlates with improvement in cardiovascular risk. Further clinical studies are required to determine if IsoPs may be useful in prognostication in CVD processes or individualization of treatment strategies.

### Malondialdehyde

MDA is generated *in vivo* via peroxidation of polyunsaturated fatty acids. MDA interacts with proteins and is itself potentially atherogenic. MDA's reaction with lysine residues generates lysine–lysine cross-links [31] which have been identified in apolipoprotein B (apoB) fractions of oxidized low density lipoprotein (OxLDL), and have been postulated to impair the interaction between OxLDL and macrophages and thereby to promote atherosclerosis [32].

MDA is typically quantified from plasma samples with the most popular method being a colorimetric assay based on the reaction between MDA and thiobarbituric acid (TBA). However, although suitable for high throughput analysis, this TBA reacting substances (TBARS) assay lacks specificity for MDA, with aldehydes other than MDA reacting with TBA to produce compounds that absorb in the same range as MDA [33]. Several ELISA kits to detect MDA are also commercially available. These antibody-based assays are typically validated against measurement of MDA by high-performance liquid chromatography (HPLC) and demonstrate good performance with improved specificity [34].

The TBARS assay has been applied as an indicator of oxidative stress in a number of cardiovascular disease models. In rats, TBARS concentrations are elevated in the plasma of streptozotocin-induced diabetic models [35–37]. Plasma TBARS concentration in these experimental models can be normalized through supplementation with various antioxidants including α-lipoic acid and aminoguanidine [36,37]. TBARS were found to be elevated in the serum of cigarette smokers [38]. A study of TBARS in 634 patients with documented coronary artery disease found that serum levels of TBARS could predict major cardiovascular events and the need for a major vascular procedure in a 3-year follow-up period independently of traditional risk factors and inflammatory markers [39]. Moreover, elevated TBARS levels predicted carotid atherosclerotic plaque progression over 3 years as assessed by carotid wall thickness on ultrasound [40]. However, a small cross-sectional study revealed no significant association between elevated TBARS and the presence of CVD after correcting for blood glucose levels [41]. Animal and human studies therefore support a potential role of lipid oxidation in predicting the progression of CVD and response to therapies.

## Oxidative protein modifications

### Nitrotyrosine

Protein tyrosine nitration is mediated by reactive nitrogen species such as peroxynitrite (ONOO^−^) and nitrogen dioxide (NO_2_), and results in a nitro group adduct on susceptible tyrosine residues [42]. Myeloperoxidase (MPO), with its transition metal centre, can react with ONOO^−^ to yield oxo-metal complexes and NO_2_ thus facilitating the nitration reaction [43]. Although the precise intermediates and mechanism for nitration *in vivo* have been a matter of controversy, measures reflective of tyrosine nitration have been used as indicators of oxidative stress. Free nitrotyrosine (3-NO_2_-Tyr) represents the turnover of nitrated proteins and can be measured by tandem mass spectrometry (MS/MS) coupled with GC or HPLC as the current gold standard technique [44]. Further studies are required to establish a normal basal range of circulating free 3-NO_2_-Tyr in healthy individuals (proposed by Pelluffo and Radi to be 1 nM) [43]. Alternatively, protein extracts from biological samples can be completely hydrolyzed before quantification of nitrotyrosines by chromatography. Results are expressed as moles of 3-NO_2_-Tyr/Tyr. Potential downside of this is that the presence of nitrite in the sample and the acid precipitation of proteins or acid hydrolysis can influence the nitration of tyrosine residues in the sample [45]. Other ways of quantifying protein nitration are immunocytochemical and immunohistochemical assays based on either monoclonal or polyclonal anti 3-NO_2_-Tyr antibodies. Antibodies against specific nitrated proteins have recently been developed and tested [46].

Nitrotyrosine formation on enzymes such as sarcoplasmic reticulum Ca^2+^-ATPase (SERCA2a) [47,48], manganese superoxide dismutase (SOD) [49], prostacyclin synthase [50], tyrosine hydroxylase [51] and aldolase A [52] inhibits their normal activity. In contrast, in the case of fibrinogen, nitrotyrosine is associated with increased activity and acceleration of clot formation [53]. Nitration of tyrosine residues in enzymes may affect their function through steric or allosteric hindrance, or gain-of-function effect as with nitrated fibrinogen (for a comprehensive review of nitration products refer to Peluffo and Radi [43]).

Nitrotyrosine formation has been observed in vascular and myocardial tissue in both healthy individuals and those with CVD [54]. In a case control study of 100 patients with established coronary artery disease (CAD), plasma protein-bound nitrotyrosine levels were found to be significantly higher among patients with CAD even after adjustment for traditional risk factors for CVD and CRP [55]. Nitrotyrosine formation on SERCA2a is significantly higher in cardiac tissue of humans with dilated cardiomyopathy compared with healthy controls [47]. Furthermore, nitration of proteins and lipoproteins may also play a direct pathophysiological role. For example, nitrated LDL is taken up by macrophages leading to foam cell formation [56].

Despite the pathophysiological role of nitration, there are several challenges in applying nitrotyrosine as a CVD biomarker. In the case of atherosclerosis, circulating nitrated proteins and lipoproteins may not accurately reflect the degree of nitration of key proteins in the vessel wall or tissue of interest [57]. Furthermore, current methods of detecting nitrotyrosine are relatively expensive and impractical for scaling up for high-throughput screening and analysis. Further studies are needed to address these issues before nitrotyrosine can be adopted as an oxidative stress biomarker ready for the clinic.

### S-glutathionylation

**Fig. 3:**
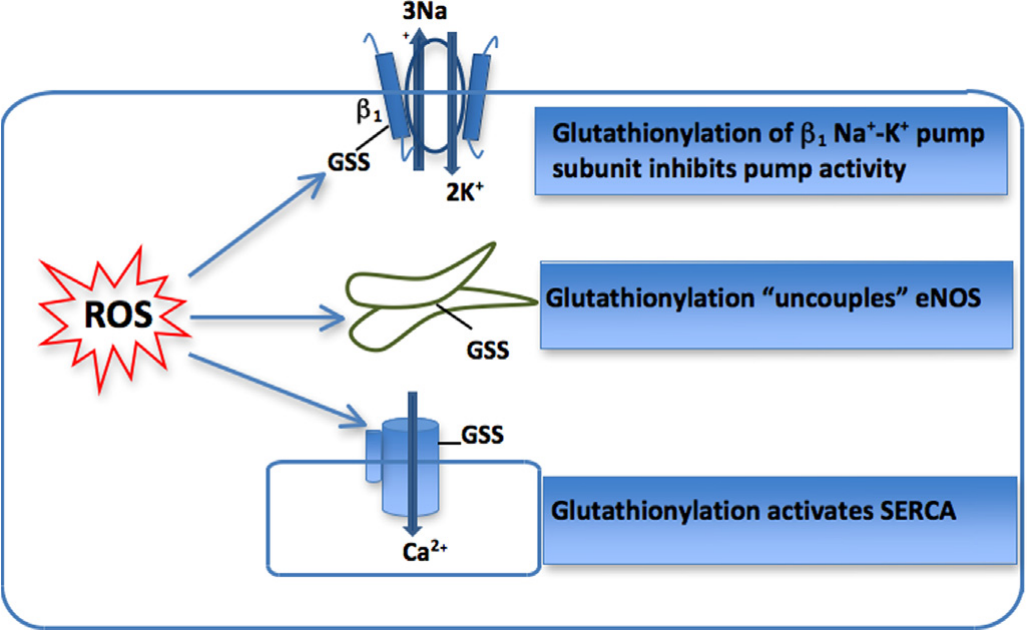
Schematic illustration illustrating the functional effect of glutathionylation of key cardiovascular proteins eNOS [58], SERCA [13], and Na^+^–K^+^ pump [10,60].

S-Glutathionylation, the formation of a disulphide bridge between a reactive cysteine residue and the abundant cellular tripeptide glutathione, is a stable yet reversible reaction that confers a 305 Da negatively charged group. This oxidative modification can exert effects on protein tertiary structure and function in a manner similar to phosphorylation [10,13] and has been shown to mediate redox regulation of a number of key cellular proteins, including endothelial nitric oxide synthase (eNOS) [58], ryanodine receptor [59], SERCA [13] and Na^+^−K^+^ pump [10,60]. The impact of glutathionylation of each of these membrane proteins has been reported in either the myocardium and/or vascular tissue – with altered function resulting in alterations in intracellular Na^+^ and Ca^2+^ handling, and other key signalling pathways particularly relevant to cardiovascular function [10,61]. However, the direct usefulness of measuring glutathionylation of these proteins as biomarkers is hampered by difficulty in accessing the tissue in which these functionally relevant modifications occur. Researchers have therefore investigated the potential of S-glutathionylation of proteins in circulating cells (e.g. erythrocytes). S-glutathionylation of haemoglobin has been proposed as a marker of oxidative stress [62], and is increased in patients with diabetes, hyperlipidaemia and renal failure [63,64]. In contrast to glutathionylation of eNOS, SERCA and the Na^+^–K^+^ pump (as illustrated in Fig. 3), the functional significance of glutathionylation of haemoglobin is not well established. The argument for the use of S-glutathionylation as a biomarker of oxidative stress would be substantially strengthened by finding a susceptible candidate protein whose function is modified in the circulating cells in parallel with modification of the same molecule in the vasculature or myocardium.

An additional challenge facing the use of glutathionyated proteins as biomarkers of oxidative stress is that measurement of glutathionylated proteins is prone to methodological artefact and requires careful specimen handling and preparation [65]. S-glutathionylation of susceptible proteins is commonly measured using low-resolution techniques such as Western Blotting under non-reducing conditions [14]. More efficient approaches include the use of MS techniques, or, potentially, ELISA with monoclonal anti-glutathione antibody (as has been developed for actin [66] and recently established by our Group for β_1_ subunit of the Na^+^–K^+^ pump [67]). Measurement of S-glutathionylation of target proteins with important functional consequences is a promising biomarker for CVD processes and merits further exploration of accurate quantification methods and predictive value for prognostication in appropriately designed prospective studies.

## Myeloperoxidase

MPO is a haeme enzyme that is abundant in granules of human inflammatory cells such as activated neutrophils, macrophages and monocytes. MPO acts as a master enzyme in the generation of a range of ROS by catalyzing the conversion of hydrogen peroxide (H_2_O_2_) to species including ^•^OH, ONOO^−^, hypochlorous acid (HOCl), and NO_2_. MPO-derived ROS can then modify lipids, lipoproteins and proteins.

MPO function can be measured by peroxidase activity assays such as the formation of guaiacol oxidation products that can be easily measured spectrophotometrically [68]. MPO mass/concentration can be quantified in biological samples using a range of commercially-available ELISA plates. Sample collection, handling and processing affects the quantification of MPO. For example, heparin in the patient or the collection tube could alter measurements [69].

MPO plays an essential role in host immune defences because of its unique ability to generate HOCl, which has potent antimicrobial activity. Since its discovery in 1994, many studies have also implicated MPO in the pathogenesis of atherosclerosis, showing that it is enriched within atheromatous plaques [70]. Inflammatory cells recruited into the vascular wall release MPO-derived ROS that can in turn promote endothelial dysfunction by reducing the bioavailability of nitric oxide [71], generate atherogenic OxLDL [72], and modify high density lipoprotein (HDL), impairing its function in cholesterol efflux [73]. Elevated circulating MPO levels have been found to be associated with the presence of CAD [1]. In prospective studies, high MPO levels were able to predict increased risk of developing CAD in healthy individuals [74]; cardiovascular events in patients presenting to emergency with chest pain [75]; and increased risk of myocardial infarction and death in patients with acute coronary syndrome [76]. The results of these large, prospective studies, as well as the ready availability of commercial assays, make MPO levels one of the most promising biomarkers of oxidative stress for clinical cardiologists [77].

## Oxidized low-density lipoprotein and oxidized phospholipids

The oxidation and glycation of LDL and phospholipids plays a central role in the pathogenesis of atherosclerosis, with the adducts being both proatherogenic and proinflammatory [78]. The oxidation of LDL can occur non-enzymatically or can be catalyzed by enzymes such as 12/15-lipoxygenase. OxLDL formation occurs primarily within vascular walls where it is taken up by macrophages via scavenger receptor pathways to form foam cells. Accumulation of OxLDL within the vascular walls also stimulates the overlying endothelial cells to produce proinflammatory cytokines including adhesion molecules such as intercellular adhesion molecule-1 (ICAM-1), vascular cell adhesion molecule-1 (VCAM-1) and endothelial selectin (E-selectin) [79].

Original studies of OxLDL depended on detection of circulating plasma autoantibodies against various oxidation-specific epitopes of OxLDL. OxLDL is now more frequently detected using specific monoclonal antibodies that directly recognize unique oxidation-specific epitopes. There are currently 3 plasma OxLDL ELISAs available for research (and not clinical) purposes (reviewed by Tsimikas [78]). The OxLDL-E06 ELISA assay quantifies oxidized phospholipids on apoB-100 molecules. The LDL-DLH3 acts in reverse to the OxLDL-E06 ELISA assay by quantifying apoB-100 on oxidized phospholipid molecules, but uses different monoclonal antibodies for detection. The OxLDL-4E6 sandwich ELISA assay detects MDA-LDL and copper oxidized-LDL epitopes, and is commercially available for experimental use. The stability of OxLDL in storage at −80° and the fact that it can be reproducibly quantified in stored samples using ELISA are technical advantages that enable its application for screening large populations [80].

OxLDL levels are higher in patients with CVD [81], and increasing OxLDL levels correlate with increasing severity of disease (e.g. stable angina vs. unstable angina vs. myocardial infarction) [82]. OxLDL levels also appear to be predictive of future CAD in apparently healthy men [83]. However, lowering OxLDL with antioxidant therapies has not been shown to decrease rates of cardiovascular events. A study of 353 healthy subjects revealed that vitamin E supplementation decreased circulating oxLDL but did not slow down the progression of carotid artery intima-media thickness over a 3-year period [84]. Thus, although promising, further studies of the clinical utility of this biomarker are required.

One of the mechanisms of protection by high-density lipoprotein (HDL) against the atherosclerotic process is by decreasing lipoprotein oxidation and generation of OxLDL. A major contributor to this antioxidant protective effect is via the HDL-associated paraoxanase (PON) which has peroxidase-like activity [85]. The failure of niacin to reduce the incidence of vascular events in the Heart Protection Study 2 despite achieving significant increases in HDL levels [86] may, at least in part, be explained by a lack of “functionality” of the HDL in regard to both its cholesterol efflux, as well as antioxidant properties (Khera A, et al. JACC, 2013; 61(10), E1390). Novel efficacious treatments therefore need to focus on targeting enhancement of the cholesterol efflux and antioxidant function of the HDL rather than simply increasing the levels.

## ROS-induced changes in gene expression

ROS levels have been shown to influence the expression of key genes involved in regulating cellular and systemic oxidative stress. A prime example is Nuclear factor (erythroid-derived 2)-like 2 (Nrf-2), a transcription factor that is upregulated in response to oxidative stress and drives the increased expression of numerous cellular antioxidant enzymes [87]. Additional examples include peroxisome proliferator-activated receptor gamma coactivator 1-alpha (PGC-1α) [88] and the thioredoxin family as reviewed by Lee and colleagues [89]. It has been proposed that profiling the expression of these ROS-sensitive genes using microarray technology may be a valuable tool, particularly relevant to assessing cardiovascular redox status. This approach remains to be explored. Low level of expression of these genes may reflect a low level of oxidative stress in the relevant system or individual variation in response and may result in a higher level of oxidative cellular damage. Whether gene expression profiling of cells in commonly collected biological samples (e.g. blood cells) accurately reflects the gene expression in cardiovascular tissue has yet to be resolved, and this presents a challenge for clinical applicability of the ROS-responsive genes as biomarkers.

## Measuring the net antioxidant capacity of the serum

Activity of antioxidant enzymes such as catalase, glutathione peroxidase 1 (GPX-1) and SOD have been quantified in plasma as measures of antioxidant capabilities. In a prospective study of patients with suspected coronary artery disease, erythrocyte GPX-1 and not SOD activity was inversely associated with incidence of cardiovascular events after adjusting for cardiovascular risk factors [90]. From a technical perspective, the enzyme activities of the GPX-1 and SOD remain stable even when the erythrocytes of the samples were haemolysed and stored frozen [90]. The commercial availability of antioxidant enzyme assay kits allows this potential biomarker to be evaluated in a large-scale high-throughput screening.

## Future directions

Significant progress has been made in primary and secondary prevention of cardiovascular adverse events, most prominently in atherosclerotic-related diseases and heart failure syndromes. Optimization of such therapies at the level of individual patients is however the “holy grail” in clinical cardiology. The burden of atherosclerotic disease, for example, in one individual is the culmination of years of ongoing insults; and successful treatment is that which has the biggest effect in halting the ongoing pathophysiology, rather than reversing the previously laid down atheroma, and ideally in stabilizing the plaque and reducing the “soft plaque” component. Quantification of both disease load and best treatment response is thus very challenging. The JUPITER trial supported the clinical utility of assessing inflammatory status in guiding intervention to limit cardiovascular events [91]. However, as ROS lie downstream from the inflammatory driver, as well as other non-inflammatory mediators of cardiovascular disease, an effective biomarker of ROS may have even greater potential.

**Fig. 4:**
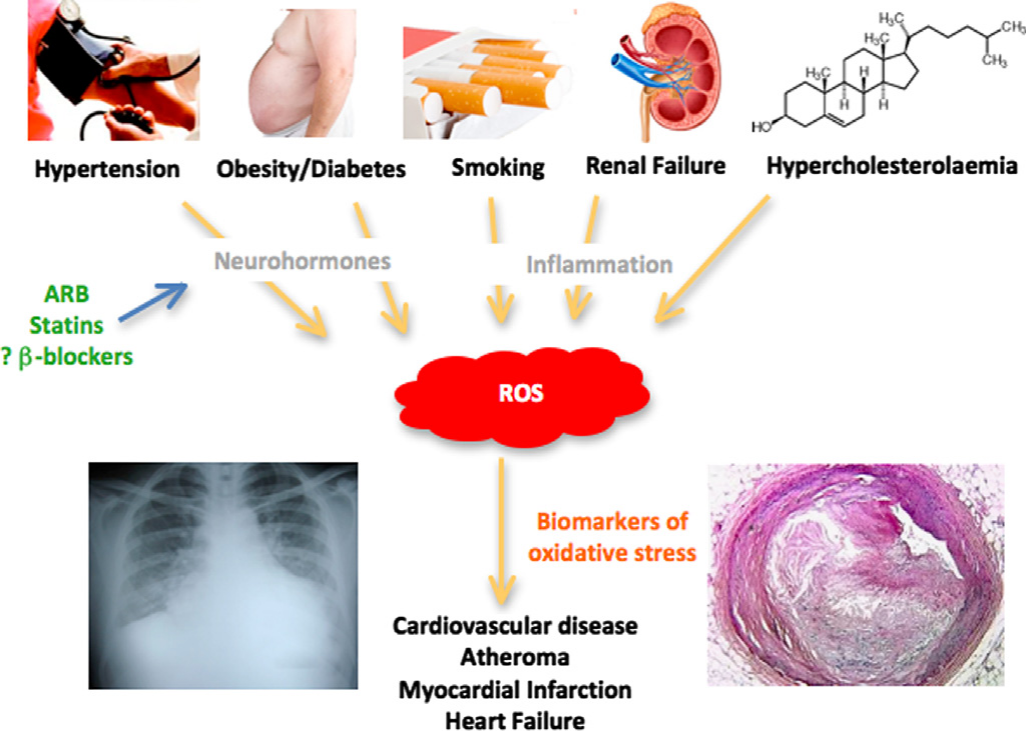
Schematic illustration of ROS as a common mediator of cardiovascular disease, making ROS-based biomarkers excellent “integrators” for total cardiovascular risk. The demonstrated effects of potent pharmacotherapies (e.g. ARB, angiotensin receptor blockers [100,101]; statins, HMG-CoA reductase inhibitors [102]; and β-blockers, β adrenergic receptor blockers [92]) on markers of oxidative stress suggest that biomarkers of ROS may be an early measure of the success of pharmacotherapy in a particular patient, and thus be a useful therapeutic guide in patients who are unable to tolerate a “cocktail” of agents.

**Fig. 5:**
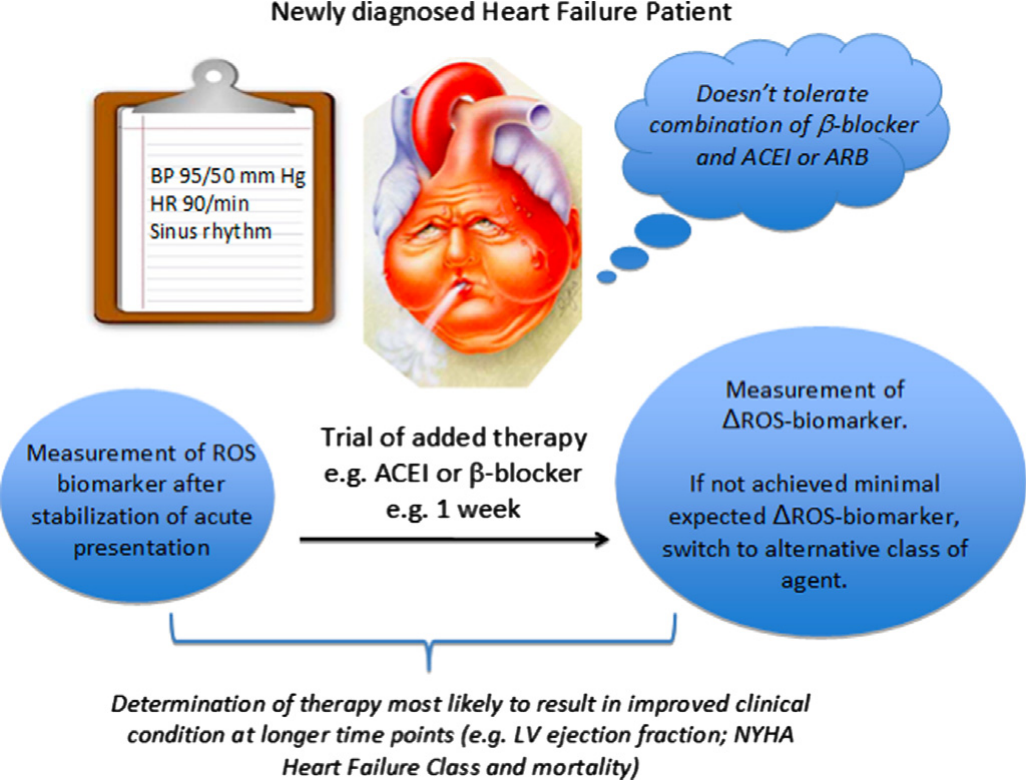
Schematic illustration of the potential application of ROS biomarker for early assessment of treatment efficacy, particularly useful for patients intolerant of combination therapies.

Effective pharmacotherapies with prognostic significance in atherosclerosis and heart failure (as well as other conditions such as diabetes and hypertension that predispose humans to CVD) exert at least some of their clinical efficacy by reducing oxidative stress and its consequences [10,11,92]. Since increased redox stress is a major paradigm in pathophysiology of these disease states, the utility of oxidative stress biomarkers in prognostication and guidance of individualized treatment is driven by their potential to act as an “integrator”, reflecting the total impact of the many pathophysiological processes (Fig. 4). A reduction in pathophysiologically-relevant oxidative biomarkers with a particular combination of pharmacological therapies (e.g. angiotensin converting enzyme inhibitors, β-blockers and statins) may provide valuable insight both into the efficacy of the treatment and guidance to selection of the most effective drugs/dose regimens for an individual patient (Fig. 5), particular in those that do not tolerate a combination “cocktail” of these proven medications. However, as ROS also play a physiological role in signalling, the relationship between a decrease in ROS biomarker and the response to treatment is not simple. The critical role of ROS in cellular and vascular homoeostasis under baseline conditions may partially explain the apparent paradox of vitamin E reducing markers of ROS, but not improving the rate of plaque progression [84]. The complex effects of ROS in physiology and pathophysiology highlight the importance of identifying the biomarker that has maximum specificity for pathophysiological effects in the relevant compartments for it to be useful for individualization of treatment strategies.

In addition to a potential utility as a guide for administration of well-established pharmacotherapies, if a biomarker of oxidative stress is shown to be of pathophysiological relevance, it may also be useful in research aimed at identifying novel treatments with antioxidant properties that can alter the disease process in a prognostically-important manner. Despite promising results in experimental and small clinical studies, large clinical trials of antioxidants have failed to significantly improve cardiovascular outcomes in a wide-range of clinical conditions [93–95]. This may result from difficulties in targeted delivery of antioxidant therapy to the key cellular microdomains [96]. One such important domain is the caveolae – home to many redox-regulated enzymes critical for cell function (including eNOS and Na^+^–K^+^ pump). If a biomarker was proven not only to be a marker of oxidative stress but also to reflect redox modifications involved in cell signalling and functional alteration of key cellular proteins, it may be useful in identification of novel effective treatments.

## Conclusion

As oxidative stress is a unifying feature of almost all of the cardiovascular risk factors known to drive the atherosclerotic process, as well as a factor that is increased in response to neurohormonal abnormalities in heart failure and in cardiac ischaemia, a biomarker that reflects oxidative stress may be criticized for its lack of specificity. However, the lack of specificity may reflect the unique ability of biomarkers of oxidative stress to integrate these risk factors and evolving CVD processes and hence be of relevance to prognostication. With technical advances in quantification of biomarkers of oxidative stress and validation in prospective clinical studies of their prognostic significance there is the potential for these to be integrated into current management schemes of cardiovascular disease. Novel biomarkers in circulation that reflect pathophysiologically relevant oxidative signalling cascades within critical cellular microdomains in cardiovascular system have the potential to supersede the currently available biomarkers. However, for this to occur, both the redox biology community and clinical researchers need to team up to design studies that go beyond just validating the association of a marker with severity of disease. The focus needs to include assessment of the prognostic ability of the marker over standard clinical measures, and the potential utility of the biomarker to tailor treatment for the individual patient and improve outcomes.

## Sources of funding

KKG is supported by a scholarship from Heart Research Australia. CCL is supported by a Fellowship (PF 12S 6924) from the National Heart Foundation of Australia. GF is supported by the University of Sydney Medical Foundation.

## Disclosures

None.

## Figures and Tables

**Table 1:** Advantages and disadvantages of various biomarkers of oxidative stress.

**Biomarker**	**Advantages**	**Disadvantages**	**Comments**	**References**
IsoPs	Can be detected in various samples (serum, urine) and has been shown to be elevated in the presence of a range of CV risk factors.	Current methods of quantification are impractical for large-scale screening (GC/MS) or requires further validation (immunoassay kits).	No evidence linking this biomarker to clinical outcomes yet.	[22,24,25]
MDA	Technically easy to quantify spectrophotometrically using the TBARS assay. ELISA kits to detect MDA also have good performance. Studies show MDA can predict progression of CAD and carotid atherosclerosis at 3 years.	TBARS assay is non-specific (can detect aldehydes other than MDA) and sample preparation can influence results	Shows promise as a clinical biomarker, however does not have a functional impact on the pathophysiology of CVD.	[33,34,39,40]
Nitrotyrosine	Human studies have demonstrated association with CAD independent of traditional risk factors	Circulating levels are not equivalent to tissue levels. Current detection methods are expensive and impractical for scaling up.	Nitrotyrosine formation on particular cardiovascular proteins have direct effect on function.	[43,55]
S-glutathionylation	S-glutathionylation of SERCA, eNOS and Na^+^–K^+^ pump demonstrated as biomarkers as well as role in pathogenesis.	Detection of S-glutationylation prone to methodological artefact.	Modified Hb currently being investigated as biomarker.	[10,13,58,65]
		Access to tissue (myocardium, vasculature) where modification occurs presents a clinical obstacle.		
MPO	Commercial assays available. Strong evidence that MPO correlates with CVD risk.	Influenced by sample storage and time to analysis.	MPO is a promising biomarker for CVD risk prediction.	[1,69,74–76,97–99]
OxLDL	Elevated in CAD, increasing OxLDL correlates with increasing clinical severity. Also is predictive of future CAD in healthy population. Good reproducibility from frozen samples.	Reduction in OxLDL by antioxidant pharmacotherapy has not been matched by reduction in CVD severity.	ELISAs for OxLDL detection readily available.	[80–83]
ROS-induced changes to gene expression	The expression of several genes may be measured simultaneously using microarray technology, potentially increasing the power of this biomarker.	Microarray technology can be manually and computationally expensive.	It is unclear if expression profiles of cells in biological samples reflect that in cardiovascular tissues.	[87,88]
Serum antioxidant capacity	GPX-1 demonstrated to be inversely proportional to CAD. Commercial kits available to measure antioxidant capacity. Reproducibly quantified despite frozen sample storage.	Antioxidant activity in serum may not reflect that of cellular microdomains that are important to the pathogenesis of CVD.	Clinical relevance of antioxidant quantification to CVD risk need further investigation	[90]
